# COVID-19: UK frontline intensivists' emerging learning

**DOI:** 10.1177/1751143720931731

**Published:** 2020-06-12

**Authors:** Hugh Montgomery, Niran Rehill, Luigi Camporota, Nicki Credland, Mike Grocott, Mark Hamilton, Daniel Martin, Brijesh Patel, Ganesh Suntharalingam, Tamas Szakmany, Shahana Uddin, Andre Vercueil, Mike Roberts

**Affiliations:** 1 158992 Whittington Hospital, 410376 Intensive Care Society [Chair], London, UK; 2 NIHR Applied Research Collaboration North Thames, London, UK; 3 Guy's and St Thomas' Hospital NHS Foundation Trust, London, UK; 4 British Association of Critical Care Nurses (BACCN), Newcastle upon Tyne, UK; 5 University Hospital Southampton NHS Foundation Trust, Southampton, UK; 6 St George's Hospital, London, UK; 7 Royal Free Hospital, London, UK; 8 Royal Brompton and Harefield NHS Foundation Trust, London, UK; 9 Northwick Park Hospital, 410376 Intensive Care Society, Harrow, UK; 10 97644 Royal Gwent Hospital, Newport, UK; 11 King's College London, London, UK; 12 King's College Hospital, London, UK; 13 154310 UCL Partners & 4617 Queen Mary University of London, London, UK

**Keywords:** Coronavirus, COVID-19, SARS CoV-2, critical care

## Abstract

The Intensive Care Society held a webinar on 3 April 2020 at which representatives from 11 of the most COVID-19 experienced hospital trusts in England and Wales shared learning around five specific topic areas in an open forum. This paper summarises the emerging learning and practice shared by those frontline clinicians.

## Introduction

**This is NOT a clinical guideline. The suggestions for practice outlined represent professional opinion only.**

At the time of writing, 51,608 people have tested positive for COVID-19 in the UK in less than three months; 5373 have died.^1^ These figures will have risen by the time you read this. Around 5%^2^ of COVID-19 infections appear to require intensive care unit (ICU) admission. As the number of people contracting the illness increases rapidly, so does ICU clinical experience. Hundreds of intensivists will by now have had to manage multiple complex COVID-19 patients.

A variety of guidelines have been rapidly produced (e.g. NICE guidance on critical care escalation,^3^ Surviving Sepsis guidelines^4^ and more), and NHS England with the Faculty of Intensive Care Medicine plan release of UK management guidelines shortly. However, solid data upon which to base these are sparse. As a result, optimal management depends on rapid dissemination of experiential learning.

To address this need, the Intensive Care Society held a webinar on 3 April 2020 at which representatives from 11 of the most COVID-19 experienced hospital trusts in England and Wales shared learning around five specific topic areas in an open forum.

This paper summarises the emerging learning and practice shared by those frontline clinicians. It represents their professional opinions at the time and should not be used as a clinical guideline document. Key emerging knowledge and suggestions for practice are presented in **bold.** *Bullets in italics outline modifications to clinical practice made by individual trusts*.

1. COVID-19 underlying patho-physiology and presentation of respiratory failure
  - **COVID-19 appears to have several phases.^5^ Management should be guided by timing of presentation in relation to the onset of symptoms to understand where in the trajectory of the disease the patient is.**
    1. **Early phase respiratory failure seems predominantly due to impaired pulmonary perfusion. Pro-coagulation leading to micro-vascular pulmonary thrombosis has been observed. Lung compliance is generally good.**
    2. **Later respiratory failure can involve acute respiratory distress syndrome and bacterial pneumonia**
  2. Mechanical ventilation
  - **Aggressive ventilation in the early phase may adversely affect later outcomes. Starting positive end-expiratory pressure (PEEP) should be lower than previously recommended. PEEP 10 cm H_2_O or less appears satisfactory for many: higher PEEP can impair pulmonary perfusion, and gains in oxygenation are often limited. CO_2_ clearance is often not greatly improved by aggressive ventilation, given the high shunt fraction/increase in physiological deadspace which is a core driver.**
  - **Consider early proning of patients to improve VQ matching**. *Examples of clinical practice*:
     *proning on admission to ICU if in early phase (predominantly perfusion) disease. It can be done irrespective of PaO2/FiO2 (PF) ratio ratio, and if response is positive, this may avoid aggressive ventilation.*
     *using cut-off PF ratio ≤16 for proning*
     *using ‘proning teams’ to manage turning, utilising non-ICU staff from around the hospital*
   • **Pulmonary vasodilatation may provide short-term benefit.** *Examples of clinical practice*:
   *Using nitric oxide in early stages – it can help but may become refractory after 96 h or so*
   *Using nebulised or IV prostacyclin – this may be helpful as part of therapeutic trial, if you are using wet ventilation circuits*
  ** • Cast formation and plugging can affect dry circuits: wet circuits *may* be beneficial.**    *Examples of clinical practice*:
   *Using wet circuits for all-COVID areas where full personal protective equipment is in use*
   *Using checklists to monitor HMEs, e.g. 12-hourly as these can fill with water rapidly – this is important in context of reduced nursing ratio. Routinely change every 24 h in any event if not needing a change before that*.
   *Use of mucolytics (e.g. N-acetyl cysteine) may be considered*.
   *Managing patients on anaesthetic machines with higher flow rates may help limit need for soda lime changes (but beware total O_2_ use limitations), zoning the machines together, educating nursing staff on use of the machines, asking anaesthetists familiar with machines to support, transferring patients out for weaning*
   • **Severe upper airway swelling in some patients may make extubation difficult.** *Examples of clinical practice*:
   *Using dexamethasone prior to extubation, having nebulised adrenaline available, with surgical airway expertise (e.g. ENT) on site and on standby*.
   *Cohorting patients ready for extubation to areas with relevant expertise and extubation protocols*
   *A mobile airway team might be an alternative*
   • **Re-intubation rates within 24–48 h seem higher than expected (up to 60%) so delaying initial extubation for longer than usual may be sensible.**
  * Examples of clinical practice*
  *Waiting 48 h past fever resolution and monitoring the inflammatory markers to ensure hyperinflammatory state is improving before attempted extubation*
  3. Antibiotics
  - **Antibiotic usage should be judicious. There are some reports of later aspergillosis and candida infections.***Examples of clinical practice*:
    1. Stopping antibiotics in COVID patients unless clearly indicated, using procalcitonin (PCT) and other inflammatory markers to monitor for bacterial infection and restarting as required*
    2. Using procalcitonin as a ‘stop’ signal to guide when to stop antibiotic use**False negative PCTs seem less of an issue than false positives in determining antibiotic use – anecdotally, rising procalcitonin has been seen in patients without evidence of bacterial infection, perhaps in relation to ‘cytokine storm’, and so a low PCT may be more helpful (true negative) than a high PCT (false positive)
  4. Fluid balance and renal support
  - **Profound pyrexia, followed by use of CPAP, may mean that patients are dehydrated.**
  - **Careful use of fluids aiming for euvolaemia may be beneficial in early phase disease – *provided that* the ventilation effort is well controlled to avoid oedema – as perfusion is a problem and prior to intensive care patients tend to have been dry.**
  - **Renal injury has been more common in UK cases than anticipated (20–35% of ICU patients). Careful attention to adequate hydration, and use of lower PEEP, may help.**
  - **Consider commencing therapeutic anticoagulation prior to haemofiltration, as filter life appears very short in its absence.***Examples of clinical practice*:
     *Using systemic unfractionated or treatment dose low molecular weight heparin and using aPTT and anti-factor Xa levels for monitoring*
    * Close partnership with renal team to manage resources*
    * Using shorter sharper diafiltrating to service machines to more than 1 person and manage filter supply*
  5. Workforce and infection control
  - **Ensure staff are comfortable in their personal protective equipment (PPE)***Example of clinical practice*:
     *Buddying for donning and doffing, potentially using medical students to observe and confirm effective practice*
  - **Promote effective use of the teams model (e.g. for resus, intubation, proning).***Example of clinical practice*:
     *Developing standard operating procedures for tasks for helpers. Resources are available including the NHS England website and BACCN which are updated daily and can be adapted for local use*
  - **Beware of ‘cognitive overload’ which can detract from getting basic ITU care right.***Example of clinical practice*:
     *Having a tactical commander on site so the clinical leads can focus on clinical tasks & provide support to the nursing and allied staff*
